# Effect of feeding a by-product feed-based silage on nutrients intake, apparent digestibility, and nitrogen balance in sheep

**DOI:** 10.1186/s40781-016-0091-7

**Published:** 2016-02-04

**Authors:** J. S. Seok, Y. I. Kim, Y. H. Lee, D. Y. Choi, W. S. Kwak

**Affiliations:** HRC, Division of Food Biosciences, College of Health and Medical Life Sciences, Konkuk University, Chung-Ju, Chung-Buk 380-701 Korea

**Keywords:** Spent mushroom substrate, By-product feed, Silage, Digestibility, Sheep

## Abstract

**Background:**

Literature is lacking on the effects of feeding by-product feed (BF)-based silage on rumen fermentation parameters, nutrient digestion and nitrogen (N) retention in sheep. Therefore, this study was conducted to determine the effect of replacing rye straw with BF-based silage as a roughage source on ruminal parameters, total-tract apparent nutrient digestibility, and N balance in sheep.

**Methods:**

The by-product feed silage was composed of spent mushroom substrate (SMS) (45 %), recycled poultry bedding (RPB) (21 %), rye straw (11 %), rice bran (10.8 %), corn taffy residue (10 %), protected fat (1.0 %), bentonite (0.6 %), and mixed microbial additive (0.6 %). Six sheep were assigned randomly to either the control (concentrate mix + rye straw) or a treatment diet (concentrate mix + BF-based silage).

**Results:**

Compared with the control diet, feeding a BF-based silage diet resulted in similar ruminal characteristics (pH, acetate, propionate, and butyrate concentrations, and acetate: propionate ratio), higher (p < 0.05) ruminal NH3-N, higher (p < 0.05) ether extract digestibility, similar crude protein digestibility, lower (p < 0.05) dry matter, fiber, and crude ash digestibilities, and higher (p < 0.05) N retention (g/d)

**Conclusion:**

The BF-based silage showed similar energy value, higher protein metabolism and utilization, and lower fiber digestion in sheep compared to the control diet containing rye straw.

## Background

Imported roughages such as rye straw, tall fescue hay, and timothy hay are expensive, however cheap, good quality silage can be developed using the feed ingredients which are abundant and easily available in many Asian countries. Previously in our laboratory the good quality by-product feed (BF)-based roughage was successfully manufactured by ensiling spent mushroom substrate (SMS), recycled poultry bedding (RPB), rice bran, and a minimal amount of straw, with added molasses and highly cellulolytic microbes [1], which were isolated from SMS [2, 3]. The silage exhibited favorable ensiling characteristics and had a higher degradability of dry matter (DM) and crude protein (CP) than do rice straw or rye straw [1].

Despite the fact that feeding BF-based silage as a roughage source to growing or fattening beef steers improved the intakes of DM and neutral detergent fiber (NDF) [4–6]. However, to our knowledge, it is unknown how rumen fermentation parameters, nutrient digestion, and N retention are affected by feeding BF-based silage in ruminants. Therefore, this study was performed to determine the effect of replacing rye straw (a conventional roughage source) with BF-based silage on ruminal parameters, total-tract apparent nutrient digestibility, and N balance in sheep.

## Methods

### Manufacture of BF-based silage

The SMS was collected fresh from a local mushroom (*Pleurotus eryngii*) farm. The original mushroom substrate consisted of 70 % sawdust, 15 % corn cobs, and 15 % rice bran. The by-product feed-based silage was manufactured as described in Kim et al. [1] at the experimental farm of Konkuk University located in Chung-Ju city in Chung-Buk province. The SMS (45 %) was mixed with RPB (21 %), rye straw (11 %), rice bran (10.8 %), corn taffy residue (10 %), protected fat (1.0 %), bentonite (0.6 %), and microbial additive (0.6 %), and ensiled in two folds of polyvinyl bags that were placed in a 40-kg capacity plastic bag for 30 d. The microbial inoculants used in this experiment were isolated and identified previously in our laboratory [2, 3], and included *Enterobacter ludwigii* KU201-3, *Bacillus cereus* KU206-3, *Bacillus subtilis* KU3, *Bacillus subtilis* KU201-7, *Saccharomyces cerevisiae*, and *Lactobacillus plantarum*. The mixture was inoculated with the strains (each added at 0.17 % [v/w] to ensure suppying 1 **×** 10^9^ cfu/g fresh biomass). *Bacillus* sp. and *Enterobacter* sp. were cultured in plate count broth (5 g casein, 2.5 g yeast extract, and 1 g/L dextrose) at 36 °C for 24 h, *Saccharomyces sp*. was cultured in yeast malt broth (0711, Difco Laboratories Inc., Detroit, MI, USA) at 30 °C for 48 h, and *Lactobacillus* sp. was cultured in MRS broth (0881, Difco Laboratories Inc.) at 36 °C for 24 h.

### Animal treatment, feeding regime and experimental design

All animal care protocols were approved by the Konkuk University Institutional Animal Care and Use Committee. Six crossbred ram lambs (a mean body weight of 55 + 2 kg) were randomly allotted to two dietary treatments: a control diet (formulated concentrate mix + rye straw) and a treatment diet (formulated concentrate mix + BF-based silage). The chemical composition of concentrate mix, rye straw, and BF-based silage is shown in Table 1. The BF-based silage contained about 4-times higher CP and lower neutral detergent fiber (NDF) than rye straw. The formulated concentrate mix was composed of 23 % corn grain, 17.5 % wheat bran, 14 % coconut meal, 10 % wheat grain, 7 % palm meal, 6% rapeseed meal, 5.8 % molasses, 5 % distillers grain, 5% corn gluten feed, 1.5 % wheat flour, 1.4 % limestone, 1.3 % tapioca, 1 % bentonite, 0.5 % dehydrate salt, 0.45 % red clay, 0.25 % calcium phosphate, 0.2 % calcium sulfate, and 0.1 % vitamin-mineral premix. Ingredients and chemical composition of diets fed to sheep are shown in Table 2. Control or treated diet was fed in equal portions in the amount of 850 g dry matter (DM, approximately 1.5% of body weight) at 07:00 and 18:00 h. The amount of diet DM corresponded to the requirements of growing ram lambs [7].

**Table 1:** Chemical compositions of feedstuffs fed to sheep^1)^

Item	Concentrate mix (%)	Rye straw (%)	BF-based silage^2)^ (%)
Dry matter	87.9	90.0	58.3
Organic matter	91.7	95.4	88.5
Crude protein	16.4	4.2	16.9
Ether extract	2.5	0.2	3.9
Neutral detergent fiber	32.7	67.7	50.6
Acid detergent fiber	19.5	41.7	38.9
Hemicellulose	13.2	25.9	11.7
Crude fiber	11.3	37.8	31.2
Nitrogen free extracts	61.5	53.3	36.6
Crude ash	8.3	4.6	11.5

^1)^On a dry matter basis

^2)^BF-based silage was by-product feed-based silage, which was composed of 45 % spent mushroom substrate, 21 % recycled poultry bedding, 11 % rye straw, 10.8 % rice bran, 10 % corn taffy residue, 1.0 % protected fat, 0.6 % bentonite, and 0.6 % microbial additive, and ensiled for 30 d

**Table 2:** Ingredients and chemical composition of diets fed to sheep^1)^

Item	Diet with	
	Rye straw	BF-based silage^2)^
Ingredient composition (%)		
Concentrate mix	60.0	60.0
Rye straw	40.0	-
BF-based silage	-	40.0
Chemical composition (%)		
Dry matter	88.7	76.1
Organic matter	93.2	90.4
Crude protein	11.5	16.6
Ether extract	1.6	3.1
Neutral detergent fiber	46.7	39.9
Acid detergent fiber	28.4	27.3
Hemicellulose	18.3	12.6
Crude fiber	21.9	19.2
Nitrogen-free extracts	58.2	51.5
Crude ash	6.8	9.6

^1)^On a dry matter basis

^2)^BF-based silage was by-product feed-based silage, which was composed of 45 % spent mushroom substrate, 21 % recycled poultry bedding, 11 % rye straw, 10.8 % rice bran, 10 % corn taffy residue, 1.0 % protected fat, 0.6 % bentonite, and 0.6 % microbial additive, and ensiled for 30 d

During the experiment, sheep always had free access to fresh water. Diets were randomly assigned to sheep at the start of the trial provided that sheep would not receive the same diet in two consecutive trials. The experiment consisted of 5 d transition, 10 d preliminary, and 7 d collection periods. The number of observations per treatment was 6. During the experiment, the animals were kept in individual metabolism crate (1.6 m × 0.5 m) that permitted separate collection of feces and urine. Daily fecal output during the collection period was dried at 60 °C. Feces were thoroughly mixed at the end of the collection period to obtain a composited sample, which was subsequently ground through a 2 mm screen prior to storage. Daily urine output was collected in plastic bottles containing 15 ml of 13.5 N H_2_SO_4_. After weighing, 2 % of the urine volume was refrigerated and bulked for the collection period.

On the last day of the experiment (d 7), 2 hours after the morning feeding, rumen fluid (250 ml) was collected with a stomach tube using an electric vacuum pump. The fluid was strained through four layers of cheesecloth prior to the determination of pH (HI9321, Hanna Instrument, Portugal). Each 5 ml of fluid was transferred to a tube containing two drops of concentrated H_2_SO_4_ and another 5 ml to a tube containing 1 ml of 25 % (w/v) of metaphosphoric acid plus 5 ml isocaproic acid for the determination of NH_3_-N and volatile fatty acids (VFA), respectively.

### Chemical analysis

Representative samples of the test feeds that supplied to the sheep were collected and stored at −20 °C. Immediately before the analysis, all the samples were dried and ground to pass through a 1-mm filter using a sample mill (Cemotec, Tecator, Sweden). The DM fraction was quantified by drying the samples at 60°C for 48 h until constant weight. The CP, ether extract (EE), and crude ash contents were determined by the AOAC method [8]. Neutral detergent fiber (NDF, heat-stable α-amylase), acid detergent fiber (ADF) were determined using the method of Van Soest et al. [9]. Ruminal NH_3_-N was determined according to Chaney and Marbach [10]. Ruminal VFA was determined according to Erwin et al. [11] using a Trace GC Ultra gas chromatograph (Trace GC ultra, Thermo, Italy).

### Statistical analysis

Data were analyzed as a completely randomized design and subjected to one-way analysis of variance (ANOVA) using the general linear model procedure. Means separation was performed using *t*-test (Statistix7, 2000) [12] and significance was declared at *p* ≤ 0.05. Data are presented as least squares means and standard errors of the means.

## Results and discussion

### Ruminal fermentation parameters

Ruminal parameters of sheep fed the different diets is shown in Table 3. The ruminal pH of both treatments was 6.6. Ruminal pH above 6.2 means adequate rumen fermentation [13]. Additionally, feeding BF-based silage did not affect ruminal acetate, propionate, and butyrate concentrations, and acetate: propionate ratio in sheep, but increased ruminal NH_3_-N (*p* < 0.05). The NH_3_-N is used as a nitrogen source by ruminal microbes which can degrade both nonstructural and structural carbohydrates [14]. It was found that BF-based silage had a higher non-protein nitrogen portion than that of rye straw [1]. In the present study, feeding BF-based silage increased dietary CP intake by 44 %, resulting in 71 % higher ruminal NH_3_-N concentration. Adequate ruminal NH_3_-N concentrations for ruminal microbial synthesis were in 5 ~ 23 mg/dl [15, 16]. The ruminal NH_3_-N concentration for the treated group were in the upper normal range. The increased ruminal NH_3_-N could increase ruminal VFA concentration [17], but feeding BF-based silage in the present study did not affect ruminal VFA concentrations.

**Table 3:** Ruminal parameters of sheep fed the different diets^1)^

Item	Diet with		SE
	Rye straw	BF-based silage^2)^	
Ruminal pH	6.6	6.6	0.1
Ruminal VFA (mM/ℓ)			
Acetate	105.6	107.5	16.1
Propionate	16.9	22.9	3.0
Butyrate	4.5	4.7	1.1
Acetate/propionate	5.9	4.7	0.7
NH_3_-N (mg/dl)	13.2^a^	22.6^b^	1.0

^1)^Means of 6 observations

^2)^BF-based silage was by-product feed-based silage, which was composed of 45 % spent mushroom substrate, 21 % recycled poultry bedding, 11 % rye straw, 10.8 % rice bran, 10 % corn taffy residue, 1.0 % protected fat, 0.6 % bentonite, and 0.6 % microbial additive, and ensiled for 30 d

^a,b^Means with different superscripts within the same row are significantly different (p < 0.05)

### Nutrients intake and digestibility

The total feed intake are shown in Table 4. Compared to feeding rye straw, feeding BF-based silage increased CP, EE, and crude ash intake (*p* < 0.05), but decreased fiber (NDF, ADF and CF), OM, and nitrogen free extracts (NFE) intake (*p* < 0.05). Apparent nutrient digestibility and digestible nutrient intake are shown in Table 5. Digestible CP and EE intakes increased and digestible DM, OM, fiber (NDF, ADF, and CF), and NFE intakes decreased by replacing rye straw with BF-based silage (*p* < 0.05). Compared to control treatment, sheep fed BF-based silage were observed to have 1.8, 2.9 and 8.8 percentage units lower apparent digestibility of DM, NDF, and crude ash, respectively (*p* < 0.05). It appeared that about 2-fold increase in EE intake induced increased EE digestibility. The decreased DM digestibility by feeding BF-based silage was attributed to the high ash content in BF-based silage. The decreased fiber (NDF, ADF, and crude fiber) digestibility was attributed to the high lignin content in the sawdust-based mushroom substrate included in the BF-based silage [1]. It seems natural that fiber in sawdust and corn cobs constituting major source in the SMS and BF-based silage would be less digested than that in rye straw. Calculated total digestible nutrients (TDN) of both diets did not alter, indicating that BF-based silage was equivalent to rye straw in energy value for sheep. In detail, TDN content of BF-based silage was estimated to be 44.1 % when TDN content of commercial concentrate mix was assumed to be normally 79.0 %.

**Table 4:** Total feed and nutrient intake by sheep fed different diets

Item	Diet with		SE
	Rye straw	BF-based silage^2)^	
Intake (g/d)^1)^			
Dry matter	850.0	850.0	0.0
Organic matter	792.2^a^	768.7^b^	0.1
Crude protein	97.9^a^	141.3^b^	0.1
Ether extract	13.5^a^	26.1^b^	0.1
Neutral detergent fiber	397.0^a^	338.8^b^	0.1
Acid detergent fiber	241.6^a^	232.0^b^	0.1
Hemicellulose	155.4^a^	106.8^b^	0.1
Crude fiber	185.9^a^	163.4^b^	0.1
Nitrogen free extracts	494.8^a^	437.8^b^	0.1
Crude ash	57.8^a^	81.3^b^	0.1

^1)^On a dry matter basis

^2)^BF-based silage was by-product feed-based silage, which was composed of 45 % spent mushroom substrate, 21 % recycled poultry bedding, 11 % rye straw, 10.8 % rice bran, 10 % corn taffy residue, 1.0 % protected fat, 0.6 % bentonite, and 0.6 % microbial additive, and ensiled for 30 d

^a,b^Means with different superscripts within the same row are significantly different (p < 0.05)

**Table 5:** Digestible nutrient intake and apparent nutrient digestibility of different diets fed to sheep^1),2)^

Item	Diet with		SE
	Rye straw	BF-based silage^3)^	
Digestible nutrient intake(g/d)			
Dry matter	575.3^a^	560.1^b^	6.3
Organic matter	553.7^a^	536.7^b^	5.8
Crude protein	67.4^a^	99.1^b^	1.1
Ether extract	10.4^a^	23.3^b^	0.6
Neutral detergent fiber	223.0^a^	180.5^b^	4.1
Acid detergent fiber	124.7^a^	113.2^b^	3.5
Hemicellulose	98.4^a^	67.3^b^	1.4
Crude fiber	91.0^a^	74.7^b^	2.3
Nitrogen free extracts	384.9^a^	339.7^b^	3.6
Crude ash	21.6	23.3	2.2
Apparent digestibility (%)			
Dry matter	67.7^a^	65.9^b^	0.8
Organic matter	69.9	69.8	0.7
Crude protein	68.8	70.1	0.9
Ether extract	76.8^a^	89.2^b^	2.8
Neutral detergent fiber	56.2^a^	53.3^b^	1.1
Acid detergent fiber	51.6	48.8	1.5
Hemicellulose	63.3	63.0	1.1
Crude fiber	48.9^a^	45.7^b^	1.3
Nitrogen free extracts	77.8	77.6	0.8
Crude ash	37.5^a^	28.7^b^	2.7
Total digestible nutrients (%)	66.7	66.6	0.7

^1)^On a dry matter basis

^2)^Means of 6 observations

^3)^By-product feed-based silage

^a,b^Means with different superscripts within the same row are significantly different (p < 0.05)

### Nitrogen balance

The N balance of sheep fed the different diets is shown in Table 6. Because of the CP content difference between rye straw (4.2 %) and BF-based silage (16.9 %), N intake significantly increased 6.9 g/d by feeding BF-based silage (*p* < 0.05). Feeding BF-based silage increased N excretion, absorption and retention by 6, 5.1, and 0.9 g/d (*p* < 0.05), respectively. But N retention, expressed % intake or % absorbed, was not different between rye straw and BF-based silage, indicating that N retention efficiency was similar between rye straw N and BF-based silage N. Normally with dietary N intake increased, N absorption and total N excretion were increased [18] as shown in this study. The high ruminal NH_3_-N could be available mostly as a nitrogen source for ruminal microbial growth, but excessive NH_3_-N would be absorbed through the rumen wall or flow into the lower digestive tract and further absorbed, resulting in increased N retention for the BF-based silage-fed sheep.

**Table 6:** Nitrogen balance of sheep fed the different diets^1)^

Item	Diet with		SE
	Rye straw	BF-based silage^2)^	
Intake (g/d)	15.7^a^	22.6^b^	0.1
Excretion (g/d)			
Fecal	4.9^a^	6.8^b^	0.2
Urinary	7.9^a^	12.1^b^	0.2
Total	12.8^a^	18.8^b^	0.3
Absorption (g/d)	10.8^a^	15.9^b^	0.2
Retention			
g/d	2.9^a^	3.8^b^	0.3
% intake	18.4	16.8	1.6
% absorbed	26.7	24.0	2.1

^1)^Means of 6 observations

^2)^BF-basedsilage was by-product feed-based silage, which was composed of 45 % spent mushroom substrate, 21 % recycled poultry bedding, 11 % rye straw, 10.8 % rice bran, 10 % corn taffy residue, 1.0 % protected fat, 0.6 % bentonite, and 0.6 % microbial additive, and ensiled for 30 d

^a,b^Means with different superscripts within the same row are significantly different (p < 0.05)

## Conclusions

The present results indicate that BF-based silage showed low fiber digestion and high N retention by sheep compared to rye straw. The BF-based silage had similar energy value to rye straw. These characteristics of BF-based silage could be used as basic data for total mixed ration formulation in ruminants. Using BF-based silage could help reduce feed cost in many countries importing hays.
